# Catastrophic Acetabular Osteolysis and Component Migration Secondary to Surgical Cup Malpositioning: A Case Report

**DOI:** 10.7759/cureus.115066

**Published:** 2026-08-24

**Authors:** Nutsa Vakhtangishvili, Randi Vorochenko, Tornike Koshuashvili, Nino Kobidze, Aleksi Sekhniashvili

**Affiliations:** 1 Medicine, Ilia State University, Tbilisi, GEO; 2 Orthopedics and Traumatology, Pineo Medical Ecosystem, Tbilisi, GEO

**Keywords:** acetabular osteolysis, component malpositioning, edge loading, periprosthetic failure, revision arthroplasty, total hip arthroplasty, trabecular titanium

## Abstract

Severe periprosthetic osteolysis and component migration present major technical challenges during revision total hip arthroplasty (THA). We report the case of a 45-year-old man who presented with a two-week history of rapidly escalating left hip pain, severe restriction of mobility, and complete inability to tolerate weight-bearing. Eight years earlier, the patient had undergone primary THA, followed three years later by a limited revision consisting of a modular head and liner exchange with retention of the original press-fit acetabular shell. Clinical evaluation revealed 2.5 cm of shortening of the left lower extremity, without localized or systemic signs of infection. Preoperative serum inflammatory markers (erythrocyte sedimentation rate (ESR), 11 mm/h; C-reactive protein (CRP), 3.1 mg/L) were within normal reference ranges, reducing the likelihood of overt periprosthetic joint infection (PJI). However, in the absence of joint aspiration and routine intraoperative cultures, low-grade PJI could not be definitively excluded. Diagnostic imaging demonstrated catastrophic acetabular osteolysis with superior-lateral migration of the component, which was formally classified as a Paprosky Type IIIA acetabular defect. At presentation, the acetabular shell exhibited severe lateral inclination (62°), representing a plausible biomechanical mechanism for edge loading and particle-induced osteolysis. Reconstruction was performed with thorough debridement, placement of three porous trabecular titanium augments with impacted morselized bone graft matrix, and multi-screw transacetabular fixation of a revision cup, restoring the anatomical center of rotation and leg length. Following an institutional protocol of six to eight weeks of protected weight-bearing, early post-discharge follow-up demonstrated restored joint stability and pain relief. This case illustrates the biomechanical consequences of severe component malpositioning and demonstrates effective reconstruction strategies for massive uncontained acetabular defects.

## Introduction

Total hip arthroplasty (THA) is among the most successful procedures in orthopedic surgery, providing substantial pain relief and restoration of function [1]. However, long-term implant survivorship depends heavily on accurate biomechanical reconstruction, optimal component orientation, and secure primary fixation [2]. Acetabular component positioning outside accepted orientation zones, specifically excessive lateral inclination (>45°) or malalignment, can disrupt normal load distribution across the hip articulation. This abnormal loading pattern leads to localized stress concentration, known as edge loading, in which peak contact stresses are transferred to the rim of the acetabular liner [3].

Edge loading generates destructive shear forces and accelerates mechanical wear, resulting in the production of substantial amounts of microscopic polyethylene and metal debris [3]. This debris triggers a macrophage-mediated inflammatory cascade, resulting in periprosthetic osteolysis, a process of progressive, debris-induced bone resorption around the implant [4]. If left unchecked, severe osteolysis compromises structural support, leading to catastrophic mechanical loosening, component migration, and massive cavitary bone defects [4].

Managing late-stage implant migration in the setting of extensive bone loss represents a significant technical challenge in revision THA. An accurate diagnostic workup requires differentiation between mechanical wear and low-grade periprosthetic joint infection (PJI), as the management strategies differ fundamentally. Although complex acetabular reconstruction techniques using porous titanium augments and bone graft matrix are established, detailed clinical reports describing the complete failure cascade, from severe cup inclination to catastrophic Paprosky Type IIIA bone loss, remain valuable for guiding reconstructive decision-making.

Here, we present a complex case of catastrophic acetabular component migration and massive bone loss associated with severe lateral cup inclination (62°), successfully reconstructed using porous trabecular titanium augments, morselized bone graft matrix, and multi-screw transacetabular fixation.

## Case presentation

A 45-year-old man presented to our orthopedic clinic with a two-week history of progressively worsening left hip pain and severe restriction of range of motion. Before this acute presentation, his baseline mobility had progressively declined, requiring specialized assistive devices for ambulation. At evaluation, he was completely unable to tolerate weight-bearing on the affected lower extremity. The patient reported that the pain had gradually increased over time without any preceding acute traumatic event or specific movement precipitating the symptoms.

The patient's surgical history was notable for a left primary total hip arthroplasty (THA) performed eight years earlier following a high-energy motor vehicle collision that resulted in complex unilateral femoral fractures and severe post-traumatic joint degeneration. Three years before the current presentation, he underwent revision THA for mechanical component instability. His past medical and family histories were unremarkable, and a review of systems revealed no chronic illnesses, fever, or chills.

Physical examination and laboratory evaluation

On physical examination, a prominent leg-length discrepancy was evident, with the left lower extremity shorter than the contralateral side. Localized tenderness was elicited over the left hip, and mechanical pain was markedly exacerbated by attempted weight-bearing. There was no associated localized warmth, erythema, or draining sinus.

Complete blood count (CBC), white blood cell (WBC) count, and serum creatine kinase levels were within normal reference ranges. Notably, inflammatory markers, including C-reactive protein (CRP) and erythrocyte sedimentation rate (ESR), were repeatedly within normal limits, reducing the likelihood of overt periprosthetic joint infection (PJI) [1] (Table 1).

**Table 1:** Preoperative laboratory evaluation. Systemic inflammatory markers (ESR and CRP) and hematologic parameters are within normal physiological limits, lowering the suspicion of infection.

Laboratory parameter	Patient value	Reference range	Status
White blood cell (WBC)	6.4 × 10⁹/L	4.0-11.0 × 10⁹/L	Normal
Hemoglobin	14.1 g/dL	13.5-17.5 g/dL	Normal
Platelets	245 × 10⁹/L	150-450 × 10⁹/L	Normal
Erythrocyte sedimentation rate (ESR)	11 mm/hr	0-15 mm/hr	Normal
C-reactive protein (CRP)	3.1 mg/L	0.0-5.0 mg/L	Normal
Serum creatine kinase (CK)	98 U/L	39-308 U/L	Normal

Radiographic and imaging evaluation

**Figure 1:**
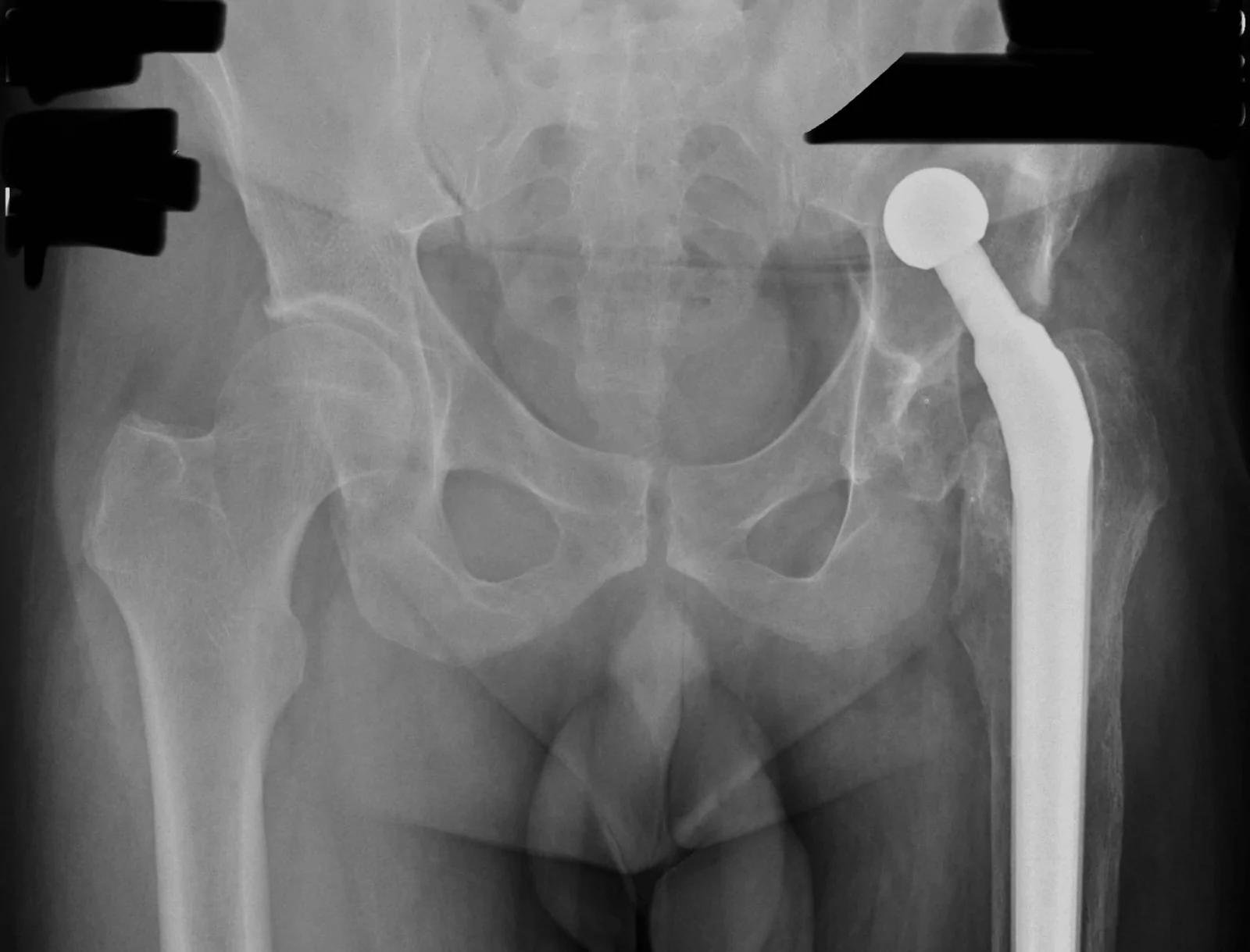
Preoperative radiographic findings. Anteroposterior pelvic radiograph demonstrating catastrophic periprosthetic osteolysis and superior-lateral migration of the left acetabular component toward the pelvic brim, accompanied by severe lateral cup inclination (62°) and marked limb shortening (2.5 cm).

Anteroposterior pelvic radiography (Figure 1) and non-contrast computed tomography (CT) demonstrated catastrophic periprosthetic osteolysis and construct failure. Particle disease resulting from microscopic wear debris activating osteoclastogenesis is a well-established cause of localized bone loss [2]. Metallosis and wear secondary to mechanical grinding were also considered, given the extent of construct destruction [3]. Imaging revealed near-complete destruction of the native acetabular floor and superior vault, with intact pelvic columns. At presentation, the acetabular component exhibited severe lateral inclination (62°) and anteversion (28°), with superior and lateral migration (>2 cm) toward the pelvic brim, inducing chronic edge loading [4].

Porous titanium augments have gained widespread application in managing such extensive loss of acetabular support [5]. Positioning components outside standard safe zones substantially alters tribological wear and contact mechanics [6,7]. The long-stem femoral component remained articulated within the displaced cup, causing superior translation of the joint center of rotation and marked limb shortening (2.5 cm).

Differential diagnosis

Periprosthetic Joint Infection (PJI)

In cases presenting with late implant migration and severe bone loss, chronic low-grade PJI must be evaluated [1,8]. In this patient, overt PJI was considered less likely based on normal systemic inflammatory markers (CRP and ESR) and the absence of constitutional symptoms [8] (Table 1). However, because preoperative joint aspiration and routine intraoperative microbiological and histological assessments were not performed, low-grade PJI could not be definitively ruled out [1].

Aseptic Loosening Secondary to Polyethylene or Metal Wear Debris

Particle disease caused by wear debris-induced osteoclastogenesis is a classic cause of localized osteolysis [2]. Although osteolysis was present, the catastrophic pattern and severe component migration pointed toward an underlying mechanical pathomechanism [2,4,6].

Metallosis and Pseudotumor Formation

Metal debris accumulation from mechanical grinding can cause soft tissue and bone destruction [3]. This was considered unlikely given the implant characteristics and the absence of palpable or radiological pseudotumor masses, as confirmed by direct intraoperative inspection.

Aseptic Loosening and Structural Failure Associated With Cup Malpositioning and Edge-Loading (Definitive Diagnosis)

Severe lateral inclination (62°) observed at presentation generated chronic edge loading [4,7] during weight-bearing. Extreme shear forces accelerated polyethylene wear, driving extensive particle-induced osteolysis, acetabular vault destruction, and component migration [2,4,6,7].

Treatment

The procedure was performed under spinal anesthesia via a posterior approach. Upon entering the joint space, there was no macroscopic evidence of purulence, hypertrophic synovitis, or tissue necrosis. Direct visualization confirmed that the acetabular shell was positioned with excessive lateral inclination [6,7].

**Figure 2:**
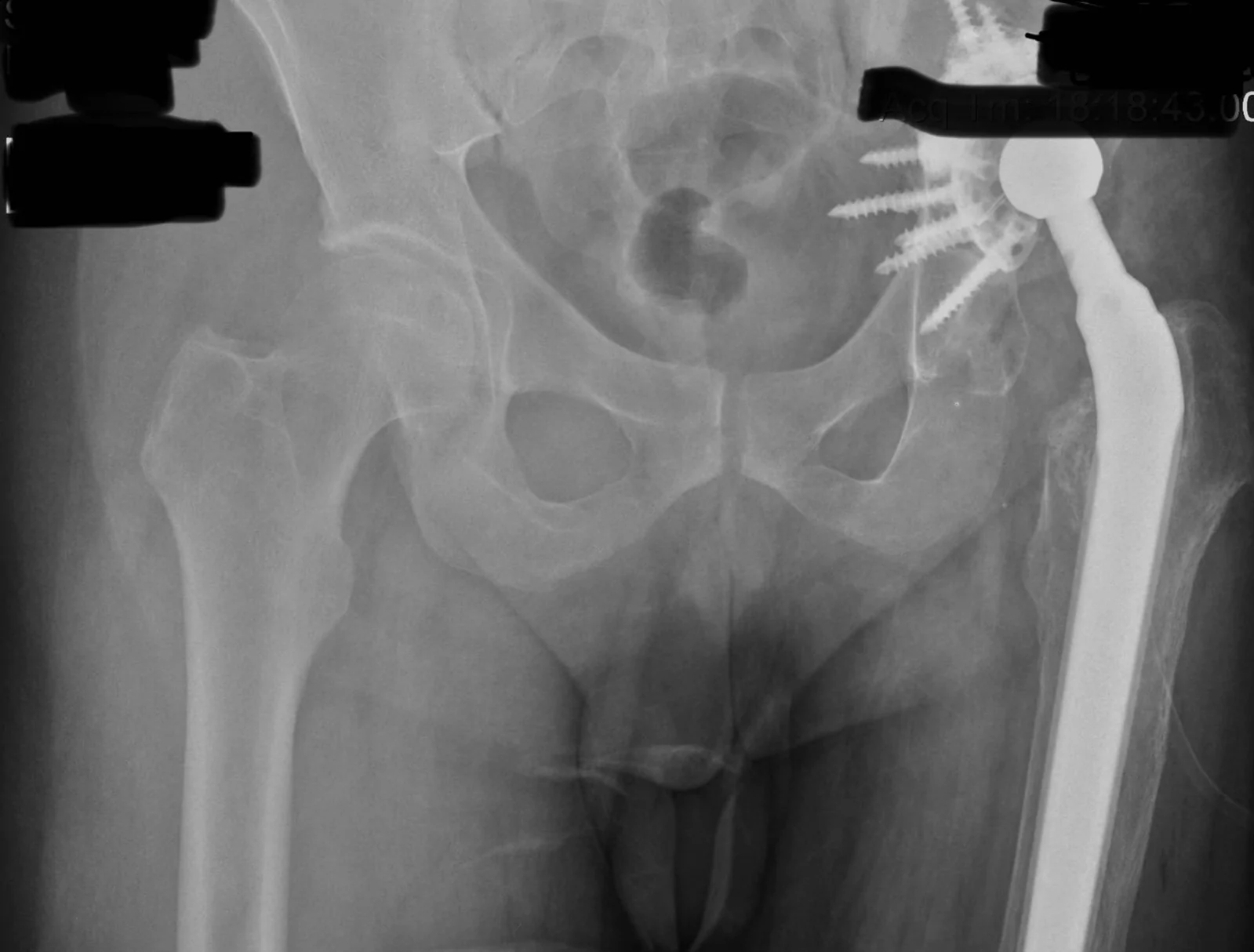
Postoperative radiographic findings. Postoperative radiograph demonstrating left acetabular reconstruction, restoring the anatomical center of rotation. Porous titanium augments and bone graft fill the vault defect, with rigid multi-screw transacetabular fixation and correction of leg-length discrepancy.

Following component extraction, extensive debridement of fibrous scar tissue was performed until bleeding bone margins were achieved. To reconstruct the uncontained Paprosky IIIA defect [9] and restore the anatomical center of rotation, three porous trabecular titanium metal augments [5,10] were impacted into the acetabular vault along with impacted morselized allograft matrix. Revision cup placement in anatomical orientation (42° inclination, 15° anteversion) was performed to correct cup orientation and minimize wear [6,7]. Rigid primary fixation was achieved using multiple transacetabular screws anchored into stable iliac bone [5,9,10] (Figure 2). Excellent joint stability and concentric reduction were verified intraoperatively.

Postoperative course and outcome

Postoperative radiography confirmed concentric joint reduction, appropriate acetabular orientation, restoration of the center of rotation, and complete correction of the 2.5 cm leg-length discrepancy (Figure 2). Postoperative pain was managed using a multimodal regimen. Standard posterior-approach hip precautions were instituted.

To protect construct integrity and facilitate osseointegration across the augment-graft interface, an institutional protocol of six to eight weeks of protected weight-bearing was initiated [11]. Early post-discharge outpatient follow-up confirmed stable implant positioning, initial pain relief, and uncomplicated wound healing. Detailed medium- to long-term follow-up was unavailable because the patient transitioned care to an outside facility following early postoperative recovery.

## Discussion

Revision THA in the setting of severe periprosthetic osteolysis and component migration presents a formidable surgical challenge. Successful reconstruction requires a clear understanding of the biomechanical mechanisms driving failure, accurate exclusion of indolent infection, and a robust reconstructive strategy that restores the anatomical center of rotation while providing immediate structural stability [1-3].

Biomechanical mechanics of edge loading

This case underscores the critical influence of acetabular cup positioning on the biomechanical longevity of total hip reconstructions. Excessive lateral inclination shifts the resultant force vector toward the superior rim of the acetabular component, inducing edge loading [4]. Edge loading dramatically increases contact pressures, accelerates wear rates, and generates severe mechanical shear stress capable of destroying adjacent native bone stock through particle-induced osteolysis [2,4]. Porous titanium metal augments have emerged as an effective reconstructive tool for managing the severe structural bone loss resulting from these failure mechanisms [5]. Positioning components outside standard safe orientation zones substantially alters wear rates and contact mechanics [6,7].

In our patient, the acetabular shell exhibited a severe lateral inclination of 62° at presentation. While cup malpositioning could reflect secondary migration due to progressive loss of bone support rather than an initial surgical execution error, this excessive inclination represents a highly plausible biomechanical mechanism for chronic edge loading and particle-induced osteolysis [2,4,6,7]. The resulting osteolytic destruction led to catastrophic collapse of the acetabular vault, superior-lateral migration of the component (>2 cm), and severe limb shortening [2].

Diagnostic differentiation of aseptic failure

In late-stage failure presenting with extensive osteolysis and superior migration, distinguishing aseptic failure from low-grade PJI is a fundamental clinical step [1, 8]. Serial measurements of inflammatory markers, specifically ESR and CRP, combined with thorough clinical evaluation, provide useful diagnostic information, as normal parameters significantly reduce the likelihood of active overt infection [8].

However, low-virulence organisms (such as *Cutibacterium acnes* or coagulase-negative staphylococci) can present insidiously without elevated serum markers [1,8]. Because preoperative joint aspiration and routine intraoperative microbiology and histology were not obtained in this case, low-grade PJI cannot be definitively ruled out [1]. This diagnostic limitation highlights the clinical necessity of routine joint sampling in late-stage migration cases to avoid misdiagnosing low-grade infection as pure aseptic failure [1,8].

Surgical reconstruction and osseointegration strategies

Management of uncontained Paprosky Type IIIA acetabular defects requires restoration of both structural volume and stable mechanical fixation [9,10]. The combination of porous trabecular titanium augments with impacted morselized bone graft matrix offers immediate structural support while providing a biomimetic scaffold conducive to biological ingrowth and long-term osseointegration [5,10].

In this case, three porous trabecular titanium augments were successfully stacked and impacted into the superior vault defect, effectively filling the cavity and re-establishing a stable platform at the anatomical center of rotation [5,10]. Multi-screw transacetabular fixation provided the immediate primary mechanical rigidity necessary for construct stability and graft incorporation [5,9,10].

Postoperative protocol and limitations

To protect the bone-implant interface during the crucial early stages of biological integration, our institution employs a protocol of six to eight weeks of protected (toe-touch) weight-bearing [11]. While immediate weight-bearing is increasingly supported in minor revisions, extended protection remains a widely accepted institutional strategy in massive Paprosky Type III reconstructions utilizing large volumes of graft and modular augments [10,11].

The principal limitation of this report is the lack of long-term postoperative follow-up, as the patient transitioned care to an outside center after initial post-discharge recovery. Although early post-discharge evaluation confirmed restored leg length, joint stability, and pain relief, long-term clinical and radiographic surveillance (>5-10 years) remains necessary to monitor for late wear, augment interface failure, or recurrent osteolysis [3,5,10].

## Conclusions

Severe acetabular component inclination (>45°) represents a major biomechanical risk factor for edge loading and destructive shear stress, which can accelerate wear debris formation and result in catastrophic periprosthetic osteolysis and component migration. In cases presenting with extensive bone destruction, evaluation for low-grade PJI using serum inflammatory markers is essential, although comprehensive joint aspiration or routine intraoperative cultures remain necessary to definitively exclude low-grade indolent pathogens. Massive uncontained bone defects, such as Paprosky Type IIIA defects, can be successfully reconstructed using porous trabecular titanium augments combined with impacted morselized bone graft matrix and multi-screw fixation, restoring the anatomical center of rotation and leg length. Following complex reconstructive procedures, an institutional protocol of protected weight-bearing for six to eight weeks helps safeguard initial fixation and promote biological osseointegration across the augment-bone interface.
